# Concentration of Municipal MBBR Effluent by FO for Resource Recovery: Batch Experiments in Side-Stream Configuration

**DOI:** 10.3390/membranes11040278

**Published:** 2021-04-10

**Authors:** Willy Røstum Thelin, Edvard Sivertsen, Gema Raspati, Kamal Azrague, Herman Helness

**Affiliations:** SINTEF Community, S.P. Andersens vei 3, N-7034 Trondheim, Norway; edvard.sivertsen@sintef.no (E.S.); gema.raspati@sintef.no (G.R.); kamal.azrague@sintef.no (K.A.); Herman.Helness@sintef.no (H.H.)

**Keywords:** nutrient recovery, resource recovery, forward osmosis, wastewater treatment, municipal wastewater

## Abstract

A novel approach for resource recovery includes forward osmosis (FO) as a concentration step in municipal wastewater treatment. The current study investigates different pre-treatment strategies including biological treatment with a moving-bed bioreactor (MBBR) at different loading rates and particle removal by filtration and sedimentation. Membrane performance and recovery potential for energy and nutrients were investigated in laboratory-scale FO experiments in batch mode using pre-treated municipal wastewater as feed and 35 g/L NaCl as a draw solution. Initial water fluxes were in the range of 6.3 to 8.0 L/(m^2^·h). The baseline fluxes were modelled to account for flux decline due to concentration effects and to enable the prediction of flux decline due to membrane fouling. Fouling-related flux decline varied from 0 to 31%. Both organic fouling and precipitation of CaCO_3_ and CaHPO_4_ were identified by using SEM–EDS. High-rate flushing resulted in complete flux recovery under most conditions. Scaling could be avoided by lowering the pH. Two operation strategies were tested to achieve this: (1) applying a bioreactor with a low organic loading rate to achieve high nitrification, and (2) adding a strong acid. A low organic loading rate and the use of additional particle removal were efficient measures that reduced organic/particulate fouling. The recovery potentials for COD and phosphorous in FO concentrate were close to 100%.

## 1. Introduction

The water sector is facing challenges that require new knowledge and innovation. Key drivers are the EU sewage sludge directive, which is to be revised and may impose new requirements on sludge management (e.g., in Norway, upcoming regulations are expected to restrict the amount of phosphorus that can be applied to farmland); the EU wastewater (WW) directive, which will also be revised and may place more focus on micropollutants; a recent EU regulation on water reuse that will take effect after June 2023; and an increased societal focus on sustainability, circular economy, digitalisation, and climate change. 

Consequently, utilities have to implement innovative solutions to deliver their services [1,2,3,4], and are setting ambitious goals: moving towards zero discharge to the air, water, and soil; creating value from waste; moving from cost coverage to profit; extracting the highest possible value from materials and resources; achieving no net-negative contribution regarding energy, greenhouse gas emissions and economy [5,6,7,8,9].

The water and wastewater treatment plants of tomorrow should, therefore, be resource recovery facilities that leave a smaller climate and environmental footprint and are capable of handling emerging compounds and future regulations on effluent quality and sludge management.

The key challenges for recovering resources from wastewater are the efficient separation of valuable compounds from WW and achieving a sufficient concentration of those for cost-efficient recovery.

Forward osmosis (FO) is an emerging membrane technology that shows significant potential for wastewater treatment with resource recovery [10,11,12,13]. One of the advantages of the FO membrane is its excellent rejection of dissolved species. Nutrients and organic matter will concentrate in the FO feed, resulting in an increased recovery potential [12,14,15,16]. However, heavy metals and emerging contaminants like pharmaceuticals, hormones, and pesticides will also be rejected to a significant extent [17,18], and measures to avoid accumulation in products such as fertilizers, must be considered.

In FO, the separation takes place due to the osmotic gradient across the membrane, without applying any external pressure. Thus, a draw solution with high osmotic potential is needed to draw water from the feed side. Seawater may be used as draw solution in coastal areas due to its abundance [19]. Because FO rejects dissolved species efficiently, the permeate will have low concentrations of contaminants and therefore be a suitable source for water reclamation. However, the recovery of water from the FO process implies that water and the draw solute must be separated by using, for example, reverse osmosis or membrane distillation [20,21,22]. These are energy-intensive technologies that need to be compared with other water reclamation solutions. 

A significant advantage of FO is its favorable anti-fouling characteristics, which means that less extensive pre-treatment will be needed compared to pressure-driven membrane processes [23,24,25]. Placing the FO-process right after the initial screening and grit removal, and combining with anaerobic treatment of the FO concentrate, will be favorable for maximizing energy recovery from municipal wastewater (MWW) [11,24]. However, biological treatment before the FO stage may also be required to reduce membrane fouling or to remove sufficient nitrogen from the total WW treatment process.

Using FO membranes in combination with biological treatment is commonly referred to as an “osmotic membrane bioreactor” (OMBR) [13,20,26]. In general, OMBRs can be categorized as submerged or side-stream configurations. In the submerged system, the FO membrane is typically placed in the final aerated stage of an activated sludge process, where aeration serves the dual purpose of providing oxygen for the bacteria and effective fouling mitigation because air bubbles aid the removal of deposited material on the FO membrane. In a side-stream configuration, the FO membrane is placed after the biological process in a separate membrane unit. A disadvantage of the side-stream configuration is that enhanced cross-flow will be necessary to control fouling, and this requires additional energy for circulation pumping. On the other hand, side-stream configurations are more flexible with respect to membrane cleaning, and can be designed to avoid limitations related to concentration build up in the bioreactor such as ammonium toxicity and impaired floc characteristics [27,28]. 

This study used FO in a side-stream configuration to concentrate municipal wastewater treated in a moving-bed biofilm reactor (MBBR) operated at different organic loading rates. Different means of particle separation, filtration, and sedimentation were used on the MBBR effluent to investigate alternative pre-treatment strategies for the FO-process. The study had the following objectives:

1. To demonstrate what membrane performance can be expected,
2. To assess flux decline due to membrane fouling using different strategies for pre-treatment,
3. To assess flux recovery by applying high-rate water flushing, and
4. To assess the recovery potential for nutrients and carbon.

## 2. Materials and Methods

### 2.1. MWW Supply and Pre-Treatment of FO Feedwater

**Figure 1:**
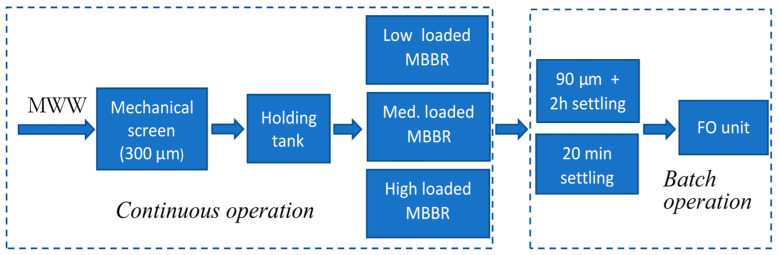
The overall experimental set-up.

The overall experimental set-up is shown in Figure 1.

Raw municipal wastewater was supplied from a residential area in the vicinity of the laboratory. The intake system consisted of an intake pump, a 300 μm mechanical screen and a storage tank. The subsequent MBBR stage included 4 continuous reactors that were operated at different organic loading rates. The characteristics of the influent to the bioreactors was: SS (72–315 mg/L), Tot–COD (207–522 mg/L), FCOD (73–192 mg/L), Tot–P (4.26–8.60 mg/L), PO_4_–P (2.47–7.72 mg/L), Tot–N (47–78 mg/L), NH_4_–N (31.64 mg/L).

The FO experiments were performed by collecting approximately 2 litres of MBBR effluent from the desired reactor. Particle removal was done by prefiltering or subsequent sedimentation. The supernatant was used as feedwater for the FO experiments. In total, 12 different FO experiments with various pre-treatment were performed as shown in Table 1. 

LL, ML and HL refers to experiments using effluent from a low-, medium- and high loaded MBBRs, respectively. Experiment ID without an asterisk refers to the most extensive particle removal using 90 μm followed by 2 h settling time. One asterisk (e.g., HL–4*) refers to 20 min settling time, and two asterisks (HL–6**) refers to 20 min settling time and pH adjustment by a strong acid.

In addition to pre-treatment, the resulting feedwater characteristics for each FO experiment also depended on fluctuations in influent WW characteristics and are given in Table S3 in Supplementary Materials, Section 2.

### 2.2. FO Lab Scale Unit

**Figure 2:**
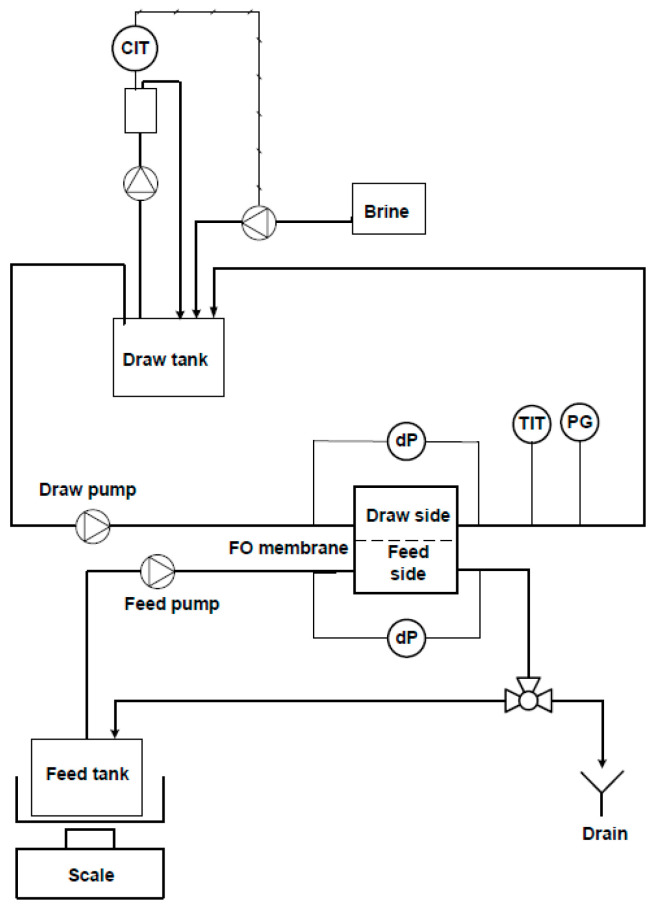
Simplified flow diagram for FO experiments performed in batch mode.

A simplified flow diagram for the lab scale FO unit is shown in Figure 2. Here, the feed side of the FO unit was operated in batch mode. Thus, the concentrate discharge from the FO membrane was circulated back to the feed tank, which was closed to the atmosphere to avoid evaporation. The mass of the feed tank was monitored by a Precision Balance ML3002T/00 from Mettler Toledo, and the permeate flux was calculated by applying a mass balance over the feed side of the FO membrane. The FO experiments were performed under isobaric conditions and ambient temperature. The calculated water flux was normalised to 20 °C to account for temperature fluctuations in the lab.

The draw solution was prepared by dissolving 35 g/L NaCl in distilled water. The concentration of the draw solution was kept constant using an 856 Conductivity Module from Metrohm that dosed NaCl brine according to a conductivity set point. 

The membrane cell had an effective membrane area of 29.4 cm^2^. A cellulose triacetate FTSH_2_O membrane was cut from A4 sheets and conditioned in distilled water an hour before assembly in the membrane cell. No spacer was used in the feed channel, whereas a standard 0.7 mm diamond spacer was used in the draw channel. The empty channel flow velocities during normal operation were 0.01 and 0.04 m/s for the draw and feed side, respectively. High-rate flushing with tap water was applied as a flux recovery measure on the feed side after each experiment using a flow velocity of 0.17 m/s.

### 2.3. Water Quality Analyses

The quality of raw wastewater, MBBR effluent, FO feed, and FO concentrate were monitored by the analyses of grab samples. Water-quality data for the different sampling locations for each of the FO experiments are given as in Tables S1–S4 in Section 2 of the Supplementary Materials. 

Inlet and effluent samples from the MBBR stage were collected to determine COD removal and nitrification for the different reactors. The FO feedwater and the FO concentrate were analysed to determine the recovery potential of phosphorous, nitrogen and carbon for each of the FO experiments being performed. Additionally, samples of the flushing solution and redissolved fouling deposits were collected for selected experiments.

ICP–MS was used to determine the elemental composition, including P, Na, K, Mg, Ca, S, Cl, Fe, Si and Al. COD, Tot–P, Tot–N, NH_4_–N, and NO_3_–N were determined by spectrometry using a Hach spectrometer. Suspended solids (SS) were determined using a GF/C filter with 1.2 μm nominal pore size and gravimetric measurement at 105 °C. Additionally, standard parameters such as pH, alkalinity, and turbidity were analysed according to standard methods.

### 2.4. Baseline-Corrected Water Flux

The observed water flux will be influenced by the flux decline from reduced driving force and membrane fouling. The observed relative flux at the end of an experiment can be calculated by Equation (1).

(1) $$\mathrm{Observed}\;\mathrm{relative}\;\mathrm{flux}=\frac{\mathrm{Observed}\;\mathrm{final}\;\mathrm{flux}\;}{\mathrm{Observed}\;\mathrm{initial}\;\mathrm{flux}}\cdot 100\%$$

The modelled baseline flux accounts for the flux decline due to reduced driving force with no influence from membrane fouling. The relative decline in baseline flux during a batch experiment is given by Equation (2).

(2) $$\mathrm{Relative}\;\mathrm{decline}\;\mathrm{in}\;\mathrm{baseline}\;\mathrm{flux}=\frac{\mathrm{Modelled}\;\mathrm{initial}\;\mathrm{baseline}\;\mathrm{flux}\;-\;\mathrm{Modelled}\;\mathrm{final}\;\mathrm{baseline}\;\mathrm{flux}\;}{\mathrm{Modelled}\;\mathrm{initial}\;\mathrm{baseline}\;\mathrm{flux}}\cdot 100\%$$

To assess fouling, it will be necessary to determine the water flux without the flux decline caused by reduced driving force. Thus, it will be convenient to use the relative baseline-corrected flux in Equation (3), which is defined as

(3) Relative baseline corrected flux=Observed relative flux+Relative decline in baseline flux

The relative flux decline due to fouling is given by Equation (4)

(4) Relative flux decline due to fouling=100%−Relative baseline corrected flux

The strategy for modelling baseline flux is provided in Section 1 in Supplementary Materials. 

### 2.5. Concentration Factors

The volumetric concentration factor ($CF_{vol}$) gives the ratio of the start volume divided by the final volume for a batch experiments as stated in Equation (5).

(5) $$CF_{vol}=\frac{V_{start}}{V_{end}}$$

The concentration factor ($CF_{i}$) for a given species can be calculated by Equation (6)

(6) $$CF_{i}=\frac{c_{end}}{c_{start}}$$

where $c_{start}$ and $c_{end}$ are the concentrations of a given species in the FO feedwater at the beginning and end of the batch experiment, respectively.

In theory, any species present in the feedwater, which is completely retained by the FO membrane and not deposited on the membrane surface, will be concentrated according to the same ratio as the volumetric concentration factor ($CF_{vol}$) in the feed tank. Thus, the relative concentration factor (*Rel* $CF_{i}$) can be used to assess the apparent recovery potential for a given specie, as shown in Equation (7).

(7) $$Relative\;CF_{i}=\frac{CF_{i}}{CF_{vol}}\cdot 100\%$$

### 2.6. Scanning Electron Microscopy

Membrane samples were analysed by scanning electron microscopy (SEM) using a Hitachi FlexSEM 1000 (Hitachi, Fukuoka, Japan) with integrated Aztec Energy EDS system. For selected FO experiments fouled membrane samples were cut and assembled in a sample holder by using carbon tape before being dried for a minimum of two days at room temperature. After drying, the samples were coated with gold before SEM was performed in low vacuum mode (30 or 50 Pa). Secondary and back scatter electron images were produced at different magnifications ranging from 200× to 5000×, to obtain morphological information about the samples. In addition, an energy dispersive X-ray analysis (EDS) was performed to obtain the elemental composition of selected areas or identified particles. To enable both the assessment of flux recovery after flushing and the characterisation of fouling deposits, two membrane cells in series were applied in some experiments where one of the cells was dismantled before flushing and SEM analyses were performed on an intact fouling layer. 

## 3. Results

### 3.1. Membrane Performance

In total 12 FO batch experiments with a duration ranging from 27 to 79 h were performed. The observed water flux during exposure to MBBR effluent is presented in Figure S2 in Section 3 in the Supplementary Materials. The initial water flux varied from 6.3 to 8.0 L/(m^2^·h), and the volumetric concentration factors at the end of the FO experiments ranged from 3.5 to 8.5.

**Figure 3:**
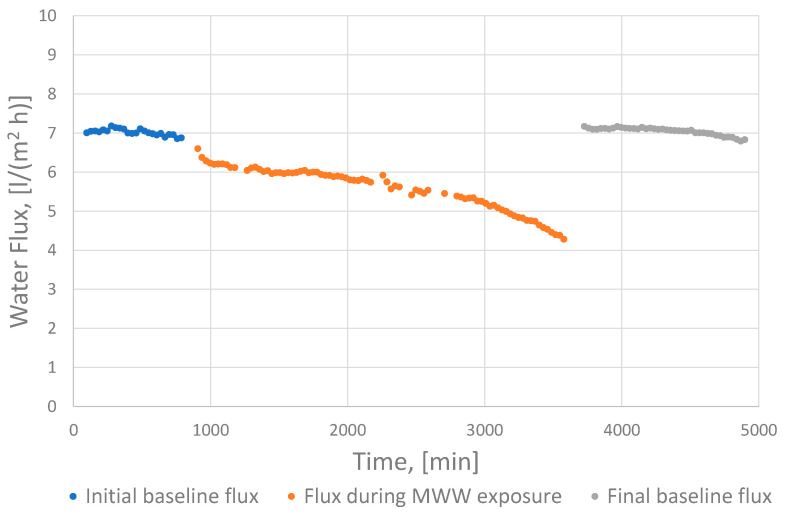
Observed water flux plotted as function of time for one of the experiments (HL–6**).

Each FO experiment included three sequences of flux measurements (Figure 3), starting with an initial baseline measurement using distilled water as feed solution (blue curve), followed by measurement during concentration of MMW (orange curve) and another baseline measurement after performing a high-rate flush with tap water for 15 min (grey curve). Figure 3 shows the progress for experiment HL–6**. 

At least part of the observed flux decline relates to the gradual decrease in the salt gradient across the FO membrane as the batch experiments elapsed. The decrease was ascribed both to reverse salt transport across the FO membrane and to the concentration of salt resulting from volume changes in the feed solution during the batch experiments. 

**Figure 4:**
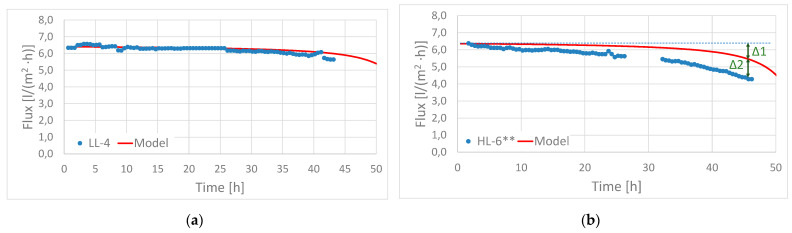
Modelled and measured water fluxes for; (**a**) experiment LL–4 with no membrane fouling and; (**b**) experiment HL-6** with significant membrane fouling.

The baseline flux was modelled for each experiment to distinguish flux decline related to fouling from flux decline related to reduced driving force. Figure 4a shows an example from experiment (LL–4) where no fouling was observed. The modelled water flux fits well with the measured flux, which was in line with the observation of a clean membrane surface with no fouling deposits after the experiment was finished. 

Figure 4b gives another example from experiment HL–6**, where significant membrane fouling was observed. The deviation between the measured flux and modelled baseline flux corresponded to the flux decline being ascribed to fouling (indicated by Δ2). The deviation between the initial and final baseline flux, indicated by Δ1, corresponded to the flux decline from reduced driving force.

**Figure 5:**
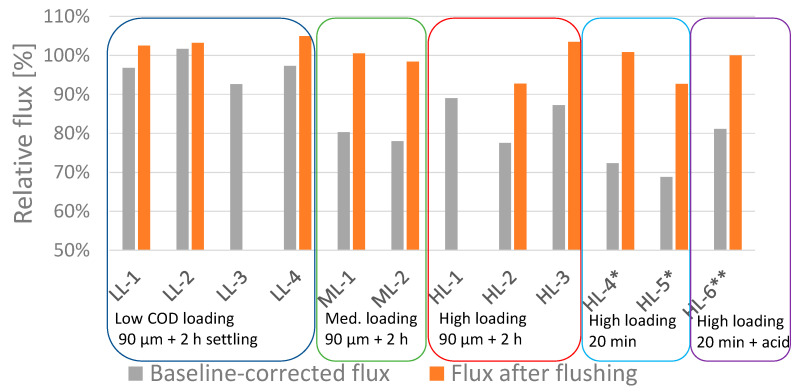
Relative baseline-corrected fluxes at the end of each FO experiment (grey bars) and relative values of pure-water flux measured after 15 min of high-rate flushing (orange bars). For two experiments (LL–3 and HL–1) high-rate flushing was not performed due to practical constraints.

Figure 5 presents the relative values for baseline-corrected fluxes at the end of each of the 12 FO experiments (grey bars). These fluxes account for the effects related to reduced driving force. Thus, the deviation from 100% is exclusively related to fouling. The experiments are grouped according to the pre-treatment applied to the FO feedwater (See Table 1). 

The introduced uncertainty in the modelling is briefly discussed in Section 4 in the Supplementary Materials. The estimated uncertainty of measured fluxes is ±3–4 %, which explains why the measured flux after flushing exceeded 100% for some experiments.

The grey bars show that no, or very little flux decline related to fouling was observed in experiments LL–1, LL–2 and LL–4. For the remaining 9 experiments, flux decline due to fouling occurred to varied extents, ranging from 8 to 31%. The most obvious observation is the positive impact of a low COD loading rate. It could also be observed that less-efficient particle removal using only 20 min settling instead of 90 μm filtration followed by 2 h settling resulted in a higher flux decline.

Figure 5 also includes the relative values of the pure-water flux measured after high-rate flushing with tap water (orange bars). The orange bars show that the initial water flux was effectively recovered for most experiments after 15 min of high-rate flushing with tap water. However, a slightly reduced recovery was observed for experiment HL–2 and HL–5*, corresponding to 93% of the initial baseline measurement. For two experiments (LL–3 and HL–1), high-rate flushing was not performed because of practical constraints.

### 3.2. Factors Influencing Fouling and Importance of Pre-Treatment Strategy

The lack of clear correlations in Figure 5 between applied pre-treatment and observed flux decline due to fouling are related to a couple of important factors. First, flux decline due to fouling will typically correlate with feedwater characteristics. However, significant fluctuations in wastewater quality over time when using real MWW, caused the characteristics of the FO feedwater to vary from one batch experiment to another even when the pre-treatment was the same. Second, the observed flux decline in the FO experiments was caused by different types of fouling.

To distinguish the effects of variation because of fluctuations in wastewater quality from those that resulted from a different pre-treatment, a principal component analysis (PCA) was performed. This was also used to explore the importance of the factors that influenced flux decline because of different types of fouling,

The variables applied in the PCA can be divided in two groups: (1) those that describe experimental conditions such as feed characteristics and choice of pre-treatment, and (2) responses observed after performing an experiment, including the parameters describing membrane performance, characteristics of FO concentrate and characteristics of fouling deposits. 

SEM and SEM–EDS were used to characterize fouling deposits on FO membranes for selected experiments. In addition, ICP–MS was applied for a more accurate determination of the inorganic composition of dissolved deposits. The results and discussion of the characterization of the fouling deposits by SEM, SEM–EDS and ICP–MS are given in Section 5 in the Supplementary Materials. In general, it was found that the membranes that were exposed to low-loading rate MMBR effluent had either no deposits or minor amounts of inorganic deposits consisting of CaHPO_4_. For the membranes exposed to medium- and high-loaded MBBR effluent, all were observed to have a relatively thick fouling layer covering the entire membrane surface. However, the composition of the fouling layer among these experiments varied considerably, ranging from mostly organic for Exp. HL–6**, to different ratios of organic and inorganic deposits, where the inorganic compounds were determined to be CaHPO_4_ and CaCO_3_. 

**Figure 6:**
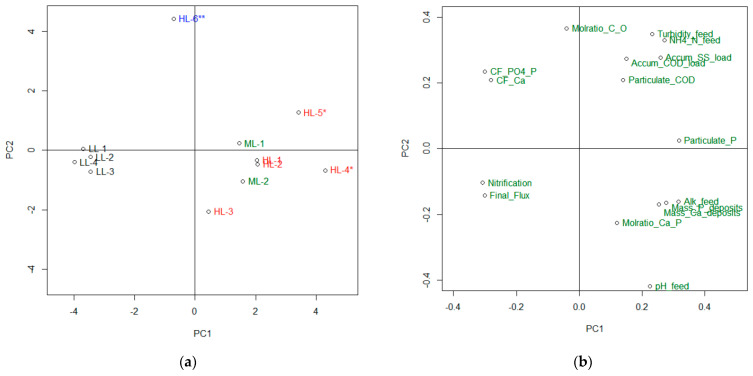
PCA based on 19 variables including feedwater characteristics, contaminant loading for filtered COD and SS, composition and characteristics of fouling deposits, concentration factors for P and Ca, and baseline-corrected water flux at the end of the experiments. The different variables are given self-explanatory short names to make the plots more readable. The cumulative explained variances for PC1 and PC2 are 64.4% and 82.9%, respectively. (**a**) Score plot; (**b**) Loading plot.

The results of the PCAs are shown in Figure 6. To enhance readability of the loading plot the variables are shown using short names. A more detailed explanation of each variable is included in Table S5 in Section 6 of the Supplementary Materials.

The score plot in panel (a) divides the experiments into three groups. The experiments performed using low-loading MBBR effluent are clustered close to PC1 to the left. Experiment HL–6**, performed with high-loading MBBR effluent adjusted to pH 5.4, is close to PC2 at the top of the score plot, whereas the remaining experiments performed with high- or medium-loading MBBR effluent without pH adjustment are scattered above and below PC1 on the right-hand side of the score plot. 

The loading plot in panel (b) illustrates the relative importance of each variable with respect to the inherent variances in the data set that are explained by PC1 and PC2. The variables contributing the most to PC1 are located at the far left or far right of the loading plot, whereas the variables contributing the most to PC2 are located at the top or bottom. Hence, variables with equal importance for PC1 and PC2 are found along the diagonals.

The loading plot shows that nitrification and final flux are positively correlated; that is, a high final flux indicating low fouling was characteristic for experiments with high nitrification, which was achieved for experiments that used low-loaded MBBR effluent. High nitrification is beneficial for two reasons: (1) it consumes alkalinity, which results in reduced pH and consequently a reduced potential for precipitation of both phosphate and carbonate-containing species; and (2) it reduces soluble COD and improves degradation of the particulate COD fraction, which is beneficial for avoiding organic fouling [29].

The variables for pH and alkalinity in the FO feed and occurrence of Ca and P in the fouling deposits being expected to correlate with inorganic scaling, are all clustered in the lower-right quadrant of the loading plot. Similarly, the variables for turbidity, SS, COD and particulate COD in the FO feed, which were expected to be correlated with organic and particulate fouling are clustered in the upper-right quadrant. Considering only PC1, all the above-mentioned variables are negatively correlated with final flux and nitrification but without any clear clustering. This makes it difficult to distinguish between organic and inorganic fouling based only on PC1. However, if the information from PC2 is included, one can distinguish among samples with different types of fouling. Samples with high inorganic fouling would be expected to have scores in the lower-right quadrant, and samples with high organic fouling would be expected to have scores in the upper-right quadrant. The interpretation of the PCA indicates that the flux decline ascribed to fouling was related to both the precipitation of scale and organic fouling to varying degrees, and that the two different types of fouling could have been discerned by combining the information in the first two PCs. 

Following the interpretation of the PCA it was shown that low turbidity, as well as low concentrations of SS and COD, in particular the particulate fraction of COD, was beneficial for avoiding organic and particulate fouling. These feed characteristics can be achieved by a low-COD loading rate in the biological stage and can be further improved by additional particle removal. Considering the range of pre-treatment strategies applied for the FO experiments, HL–4*, HL–5* and HL–6** were expected to be the most exposed to organic/particulate fouling, whereas LL–1–LL–4 were expected to have the most efficient pre-treatment to avoid organic/particulate fouling. This was in line with the flux decline observed for these experiments. 

Scaling can be avoided by lowering the pH either by using a low-loaded MBBR to achieve high nitrification or by dosing a strong acid as was done for experiment HL-6**. Experiments ML–1, ML–2, HL–1, HL–2, HL–3, HL–4* and HL–5* that were performed with low nitrification and without pH adjustment were all expected to suffer from scaling. This was confirmed experimentally. HL–4* and HL–5* were observed to have the highest flux decline amongst all the experiments. This is in line with the fact that these experiments had unfavorable pre-treatment strategies both with respect to organic/particulate fouling and precipitation of phosphate and carbonate scale. 

Figure 6 also shows that significant differences in flux decline were observed among the different experiments performed with medium- or high-loaded MBBR effluent followed by the most efficient particle removal (90 μm filtration + 2 h settling). In general, these differences were due to variations in the MWW characteristics for different experiments, and as a result the extent of organic/particulate fouling and scaling varied as well (e.g., HL–3 had a final baseline-corrected flux of 87% compared to only 78–80% for ML–1, ML–2 and HL–2). The explanation for this is the low organic fouling potential for HL–3 (low turbidity and SS in the FO feed), and low scaling potential because of a combination of low feed concentration of PO4–P and a low volumetric concentration factor at the end of the experiment. 

### 3.3. Resource Recovery from FO Feed

#### 3.3.1. Recovery Potential for Phosphorous in FO-Feed

The recovery potential of a species can be assessed using the relative concentration factor, which can also provide information on precipitation. If the relative concentration factor is 100%, there are no losses through the FO membrane and the recovery potential in the FO stage will be 100%. If the relative concentration factor is lower than 100%, there may be permeation through the FO membrane and the recovery potential will be lower than 100%. However, a relative concentration factor lower than 100% can also be caused by precipitation on the FO membrane or in the bulk stream. In such cases, the recovery potential is not necessarily affected. For example, if the fouling is reversible, the deposited matter can be recovered in a subsequent cleaning step. 

**Figure 7:**
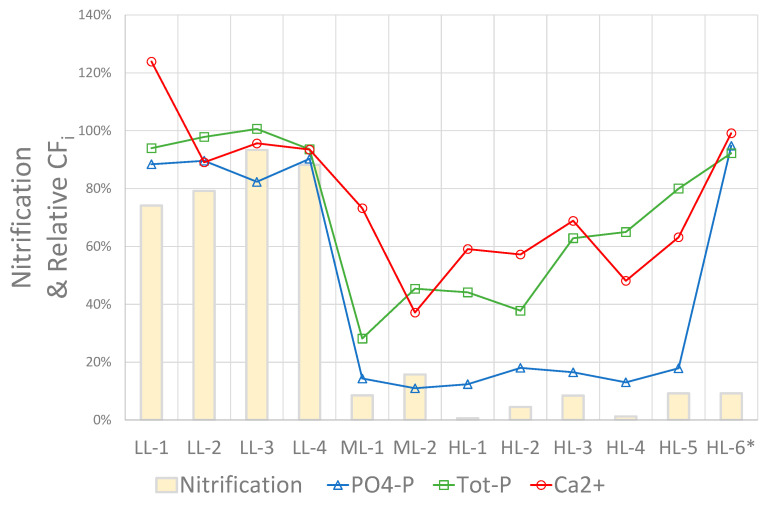
Concentration factors relative to the volumetric concentration factor for PO4–P, Tot–P and Ca^2+^, and nitrification (% ammonia converted to nitrate) in MBBR effluent.

The relative concentration factors for PO4–P, Tot–P and Ca^2+^ for the 12 FO experiments are shown in Figure 7. It was observed that the relative concentration factor for PO4–P was high (82–95%) for experiments LL–1–LL-4 and HL–6**, while it was significantly lower for the remaining experiments (11–18%). The same trend was observed for Tot–P; however, here the relative concentration factors were even higher for LL–1–LL–4 and HL–6** (92–100%). A similar trend is observed also for Ca^2+^.

The results showed that a recovery potential for phosphorous in the FO-feed close to 100% might be achieved and could be explained by the high rejection of PO4–P by the FO membrane (>99%) as several authors have reported [12]. The difference between the relative concentration factor for PO4–P and Tot–P was due to particulate phosphorus, both biologically bound phosphorous and eventually precipitated PO4–P. The low relative concentration factor for PO4–P and Ca^2+^ observed in some of the experiments indicated significant precipitation of phosphorous and calcium, but precipitation does not necessarily affect the potential for recovery. However, it may contribute to flux decline, so maintaining PO4–P in a dissolved state may simplify recovery.

#### 3.3.2. Conditions for Optimizing Phosphorous Recovery

Figure 7 also includes the nitrification efficiency in the MBBR at the time the feedwater for the FO experiments was collected. It was observed that the four experiments labelled LL (low loaded MBBR) all had high nitrification values and high concentration factors for PO4–P. A SEM–EDS-analyses of these membranes (data in the Supplementary Materials) demonstrated either very limited precipitation of calcium phosphate or no precipitation at all. The other experiments labelled ML (medium-loaded MBBR) and HL (high-loaded MBBR) were all observed to have low nitrification values. With the exception of HL-6**, the experiments labelled ML and HL also had low concentration factors for PO4–P. The explanation relates to how nitrification affects the water chemistry. Both carbonate and phosphate scales are strongly pH dependent, and precipitation is more likely at higher pH values. Since nitrification consumes alkalinity, the pH will drop during nitrification and the resulting lowered pH in the FO feed prohibits precipitation. For the experiments with FO feedwater collected from low-loaded MBBRs (LLs), the pH was 6.3–7.2, and alkalinity 0.2–0.8 meq./L compared to a pH of 7.8–8.0 and an alkalinity of 5.8–7.1 meq/L for experiments performed with medium- or high-loaded MBBR effluent. The different behavior observed for HL–6** related to the lowering of the feedwater pH from 7.9 to 5.4 by the addition of hydrochloric acid, which resulted in a decline in alkalinity from 7.1 to 1.0 meq./L.

#### 3.3.3. Recovery Potential of Nitrogen and COD

**Figure 8:**
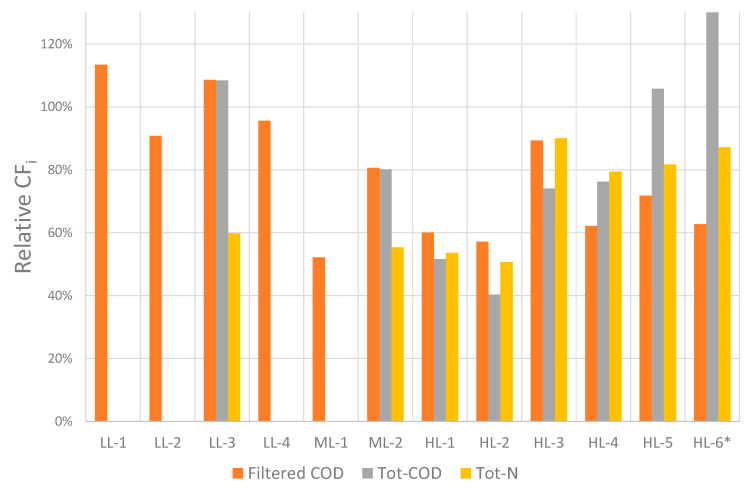
Concentration factors relative to the volumetric concentration factor for filtered COD, Tot–COD and Tot–N.

The relative concentration factors for filtered COD, particulate COD, and Tot–N are shown in Figure 8. It was observed that the relative concentration factors for filtered COD varied from 50 to >100%, and for Tot–COD from 40 to >100%. COD was not expected to permeate the membrane to a significant extent. This was reflected in its concentration factors being close to 100% for the experiments performed with low-loaded MBBR effluent, where no significant organic/particulate fouling were observed. It can be argued that concentration factors for filtered COD and Tot–COD below 100% relate to COD fouling of the membrane. 

It should be noted that the uncertainty in the Tot–COD measurements might be large for some samples because of the difficulties in collecting representative samples from small volumes with high particulate content. Thus, the Tot–COD measurement for HL–5* and HL–6**, where significant fouling was observed, is believed to be incorrect.

The characterization of fouling deposits for selected experiments indicated that the deposits did not contain nitrogen to a significant extent. Thus, the observation of a relative concentration factor for Tot–N in the range of 53–87% indicated that nitrogen was lost due to permeation through the membrane. Both nitrate and ammonia permeated the FO membrane, and the potential recovery of nitrogen was therefore lower than 100%.

## 4. Discussion

The observed initial water fluxes were in the range of 6.3 to 8.0 L/(m^2^·h). The water flux at inlet conditions in a potential future full-scale facility using the same type of membrane and seawater as a draw solution is expected to be in the same range, depending on temperature and TDS. However, since the osmotic gradient will be gradually reduced along the FO element, the average observed water flux will be somewhat lower. To increase water fluxes beyond 6–8 L/(m^2^·h) would require either (1) improvements in the performance of commercially available FO membranes, or (2) the use of draw solutions with a higher osmotic potential than that of seawater.

The highest concentration factor achieved without observing significant flux decline due to fouling was around 8. However, the concentration factor in the batch experiments was limited because of the minimum volume needed to perform water-quality analyses for the FO concentrate. The achievable concentration factor in a larger scale plant with continuous operation could, therefore, possibly be higher. However, membrane fouling, and the precipitation of inorganic species would need to be considered.

The flux decline ascribed to fouling was found to vary from 0 to 31% among the different FO experiments. In line with previous studies, the flux recovery by high-rate flushing with tap water was found to be efficient [24]. A flux recovery of 100% was observed except in two experiments. As flux decline related to fouling is typically progressive, the lower flux recovery found for two of the experiments could likely be improved by initiating flux recovery measures at an earlier stage.

The fouling-related flux decline observed for the FO experiments was ascribed to organic fouling and the precipitation of CaCO_3_ and CaHPO_4_. The development of organic fouling was promoted by high turbidity and a high concentration of SS and COD in the feedwater, especially the particulate COD fraction, which indicated that particulate fouling was more important than biofouling from microbial growth. 

Precipitation is dependent on feed concentration of precipitating species, the volumetric concentration factor, pH, alkalinity, ionic strength, and temperature. Prediction models for scaling potential should be based on the modelling of the saturation index for relevant species. However, to enable assessment of conditions at the membrane surface it will be necessary to integrate models for the calculation of saturation indexes into a transport model. 

Two operational strategies to avoid scaling during the concentration of MWW were demonstrated:

1. Applying MBBR effluent from reactors with a sufficiently low organic loading rate to achieve high nitrification, thereby reducing alkalinity and pH and consequently achieving a lower scaling potential for both CaCO_3_ and CaHPO_4_;
2. Reduce pH sufficiently to avoid CaCO_3_ and CaHPO_4_ scaling by dosing a strong acid.

For the first option, a nitrifying bioreactor was included in the pre-treatment line to lower the pH. To achieve high nitrification, a low organic loading rate was required, which also resulted in the consumption of biologically degradable organic matter and reduced the organic fouling potential. 

The second strategy used acid to control scaling. This provided flexibility in the design of the pre-treatment line. The current study indicated a faster flux decline for experiments performed with less-extensive particle removal in the FO feed, and vice versa. With respect to flux recovery, no significant differences were observed. However, long-term experiments will be needed to determine optimal pre-treatment.

The maximum concentration factor of submerged OMBRs will in practice be limited because of ammonium toxicity and floc instability [27,28]. The side-stream configuration was not influenced by these factors. However, side-stream OMBRs based on activated sludge will be needing a sludge separation stage before the FO stage to maintain the sludge concertation in the activated sludge reactor. This will not be needed if a biofilm process is used. However, particle removal prior to the FO stage may still be required to optimize FO performance. Thus, future research should determine the maximum concentration factor that can be achieved for a OMBR in a side-stream configuration at various pre-treatment and feed characteristics without precipitation taking place. 

Further studies should be performed in continuous mode to better mimic the conditions that will apply in a full-scale OMBR installation. Long-term experiments are required to assess how the selection of pre-treatment and the efficiency of different flux recovery measures influence flux decline, fouling reversibility, and recovery potential.

## 5. Conclusions

1. Demonstration of membrane performance:
  - The observed initial water fluxes when using a 35 g/L NaCl draw solution were in the range of 6.3 to 8.0 L/(m^2^·h).
  - The flux declines due to fouling beingobserved at volumetric concentration factors of 3.5 to 8.5 were in the range of 0 to 7% for experiments performed with low-loaded MBBR effluent and in the range of 11 to 31% for experiments performed with high-loaded MBBR effluent.
2. Assessment of the impact of pre-treatment strategy with respect to membrane fouling:
  - The observed flux decline due to fouling was caused by a combination of organic/particulate fouling and the precipitation of CaHPO_4_ and CaCO_3_. The importance of the different forms of deposits varied among the different experiments as a result of different feed characteristics and operating conditions.
  - Lowering of pH was important for avoiding precipitation of the phosphate and carbonate scale. Two feasible pre-treatment strategies for pH reduction were demonstrated; (1) operating the MBBR stage at a low loading rate to achieve high nitrification and (2) adding a strong acid.
  - Reducing the concentration of organics and particles in the feedwater was important for reducing organic/particulate fouling. The operation of the MBBR stage at a low loading rate was demonstrated as one means of reducing the potential for organic/particulate fouling. In general, more efficient particle removal applied upstream the FO will be beneficial for reducing organic particulate scaling.
3. Assessment of recovery potential:
  - This study confirmed that the recovery potential of phosphorous and COD in the FO-feed can be close to 100%, which is in line with previous studies [12,30]. However, further studies are required to assess the actual recovery of energy and nutrients, which must be obtained by the further processing of the FO concentrate.
4. Assessment of high-rate flushing as a flux recovery measure:
  - High-rate flushing with water was found to fully recover the initial water flux at most conditions.

## Figures and Tables

**Table 1:** Applied Pre-Treatment for FO Experiments.

Experiment ID	MBBR Loading Rate	Particle Removal	pH Adjustment
LL–1	1.2	90 μm + 2 h settling	No
LL–2	0.7	90 μm + 2 h settling	No
LL–3	0.5	90 μm + 2 h settling	No
LL–4	0.5	90 μm + 2 h settling	No
ML–1	4.8	90 μm + 2 h settling	No
ML–2	3.0	90 μm + 2 h settling	No
HL–1	5.8	90 μm + 2 h settling	No
HL–2	7.1	90 μm + 2 h settling	No
HL–3	6.5	90 μm + 2 h settling	No
HL–4*	10.0	20 min settling	No
HL–5*	15.4	20 min settling	No
HL–6**	14.2	20 min settling	Yes (HCl)

## Data Availability

The data presented in this study is available on request from the corresponding author.

## Supplementary Materials

The following are available online at https://www.mdpi.com/article/10.3390/membranes11040278/s1, Table S1: Characteristics of raw wastewater, Table S2: Characteristics of MBBR effluent, Table S3: Characteristics of FO feed, Table S4: Characteristics of FO concentrate Table S5: Explanation of short names of variables used in PCA; Figure S1: Concentration profile in FO illustrating the four transport zones, i.e., the two diffusion films on the surfaces of the membrane, the support membrane, and the membrane skin; Figure S2: Water flux as a function of volumetric concentration factor measured during period with exposure to M;BBR effluent. Measured fluxes were temperature normalized to 20 °C. The experiments are labelled according to the design value for the biological loading rate of the MBBRs providing the feed water to each of the experiments. LL means low biological loading rate, ML means medium loading rate, and HL means high loading rate; Figure S3. SEM images of fouled membranes from selected FO experiments. (a) Backscatter image at 45× magnification of membrane sample from Exp. LL-3; (b); Backscatter image at 200× magnification of membrane sample from Exp. HL-6** (c) Backscatter image at 200× magnification of membrane sample from Exp. HL-5*; (d) Secondary electron image at 5000× magnification of membrane sample from Exp. HL-4*; Figure S4. SEM-EDS analyses of deposits on membrane from Exp. HL-5*. (a) Calcium signal; (b) Phosphorous signal; (c) Oxygen signal; (d) Carbon signal; (e) Backscatter image at 2000× magnification; and (f) relative molar composition [mol%]; Figure S5. Molar ratios for carbon/oxygen and calcium/phosphorous obtained by EDS-analyses on fouled membranes from FO experiments. For LL-4 and HL-6** the Ca/P ratio was not obtained due to insignificant content of inorganic material; Figure S6. (a) Mass of P and Ca obtained from ICP-MS of dissolved fouling deposits. 100% refers to the sample with highest mass (HL-4*); (b) Molar Ca/P ratio obtained from ICP-MS of dissolved fouling deposits and SEM-EDS of fouled membranes, respectively.
