# Supplementary material for: Concentration of Municipal MBBR Effluent by FO for Resource Recovery: Batch Experiments in Side-Stream Configuration
Source: Membranes (Basel). 2021 Apr 10;11(4):278. doi: 10.3390/membranes11040278 (PMC8068858; doi:10.3390/membranes11040278)
Supplement: Supplementary file 1 [file membranes-11-00278-s001.pdf]

membranes

Concentration of municipal MBBR effluent by FO for resource recovery: batch experiments in side-stream configuration

Willy Røstum Thelin [1,*], Edvard Sivertsen [1], Gema Raspati[1], Kamal Azrague[1], and Herman Helness [1]

[1] SINTEF Community, S.P. Andersens vei 3, N-7034 Trondheim, Norway; willy.thelin@sintef.no
* Correspondence: willy.thelin@sintef.no; Tel.: (+47 92268433)

1. Transport model

The mass transport in FO can be modelled by using the mass balance for salt in each of the four different transport zones as a starting point, ref. Figure S1.

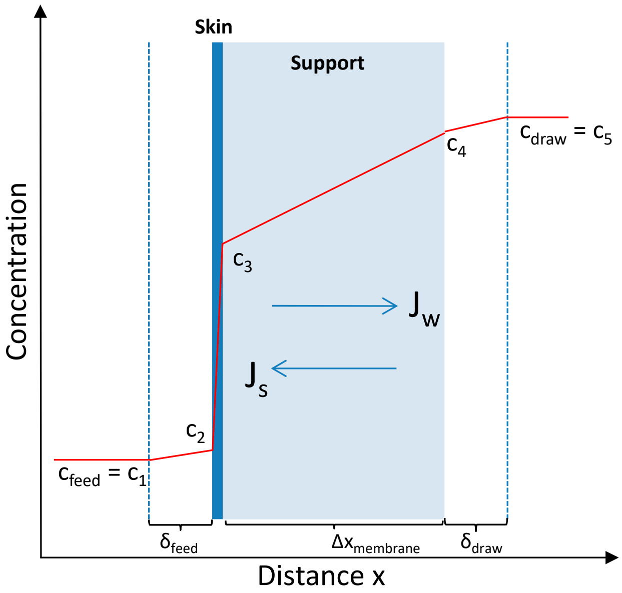

Figure S1 Concentration profile in FO illustrating the four transport zones, *i.e* the two diffusion films on the surfaces of the membrane, the support membrane, and the membrane skin.

The mass balance for salt can be described by the general equation (eq. (1)) [30]:

$$-J_s = D\frac{\phi}{\tau}\frac{dc}{dx} - J_v c \quad (1)$$

where J_s is the salt flux, J_v is the volume flux, c is the salt concentration at a given distance x, and D is the diffusion coefficient of the salt. The porosity ϕ and the tortuosity τ are given characteristics of the support membrane. Both parameters are equal to 1 in the diffusion films.

By integrating the Eq. 1 and applying appropriate boundary conditions for FO mode in each of the four transport zones, it can be shown that the concentration difference over the skin can be expressed by eq. (2) [30-32].

$$\Delta c_{skin} = c_3 - c_2 = \frac{(c_{draw} - c_{feed})\cdot e^{\left(\frac{(S+\delta_{draw}+\delta_{feed})\cdot Jw}{D}\right)}}{e^{\left(\frac{(S+\delta_{draw})Jw}{D}\right)} + \frac{B}{Jw}\cdot\left[e^{\left(\frac{(S+\delta_{draw}+\delta_{feed})\cdot Jw}{D}\right)} - 1\right]} \quad (2)$$

where c_{draw} and c_{feed} are the bulk concentrations on the draw and feed side, δ_{draw} and δ_{feed} are the thickness of the respective diffusion films, and S is the structure parameter defined as eq. (3).

$$S = \frac{\tau}{\phi}\Delta x_{mem} \tag{3}$$

The water and salt flux can subsequently be calculated at a given feed condition by inserting Eq. 2 into the respective flux equations (Eq. 4 and 5) [30].

$$J_v = A(\Delta\pi_{skin} - \Delta p) \tag{4}$$

$$J_s = -B\Delta c_{skin} = -B(c_3 - c_2) \tag{5}$$

where A and B are the permeability constants for water and salt, respectively. Δp is the transmembrane pressure, $\Delta\pi_{skin}$ is the osmotic pressure difference across the membrane skin. The osmotic pressure can be related to concentrations by the van't Hoff relationship, eq. (6).

$$\pi = iRTc \tag{6}$$

where R is the ideal gas constant, T is the absolute temperature, c is the osmolality of the solution, and i is a constant adjusting for deviations from ideal solution.

1.1 *Approximation of osmotic potential in FO feed*

The feed water used for the FO experiments contained various electrolytes. Thus, the concentration of main ions (Na^+, K^+, Mg^{2+}, Ca^{2+}, Cl^-, SO_4^{2-}, HCO_3^-, HPO_4^{2-}) were estimated based on elemental composition determined by ICP-MS analyses. To simplify the modelling of the experiments the ionic strength I of the feed water was calculated by use of Eq. 7 and subsequently converted to equivalent NaCl concentrations.

$$\mathrm{I} = \frac{1}{2}\sum_{i=1}^{n} c_i\, z_i^2 \tag{7}$$

where c_i is the molar concentration and z_i is the valence of electrolyte *i*.

1.2 *Modelling of feed concentration*

Since the FO experiments were operated in batch mode, the feed concentration during an experiment was changing as a function of time. The feed concentration ($c_{feed}(t)$) was modelled by applying a mass balance over the system according to eq. (8).

$$c_{feed}(t) = \frac{n_0 + (J_s \cdot t \cdot A_{membrane})}{V_0 + (J_v \cdot t \cdot A_{membrane})} \tag{8}$$

where t is the elapsed time from start of the experiment, $A_{membrane}$ is the membrane area, and n_0 and V_0 are the initial amount of NaCl (mol) in the feed, and the initial total volume of the feed, respectively.

1.3 *Modelling of baseline flux*

The baseline flux is defined as the pure water flux accounting for flux decline related to reverse salt diffusion and concentration effects. The baseline flux was modelled as a function of time by applying a modified version of a two-dimensional transport model for water and salt transport in osmotic membranes, which are presented previously [31]. Instead of modelling in the longitudinal

direction the mass transport was modelled for inlet conditions in the lab cell for n time steps by applying the following two generic steps:

1. Calculate the water and salt fluxes by using Eqs. 2, 4 and 5 for c_{feed} at t=0.
2. Estimate a new c_{feed} for the flux calculation in the next time step by using Eq. 8. The procedure was repeated for n timesteps corresponding to the time at the end of each FO experiment.

2. Water quality data

Table S1. Water quality data for raw wastewater collected at the day of start-up for each experiment. The unit is mg/l for all parameters.

Experiment	SS	Tot-COD	F-COD	Tot-P	PO4-P	Tot-N	NH4-N
LL-1	127	494	146	8,60	6,14	n.a.	64
LL-2	134	n.a.	130	n.a.	7,72	n.a.	41
LL-3	80	327	154	6,86	5,08	74	51
LL-4	125	359	161	8,50	5,18	78	60
ML-1	154	n.a.	134	17,10	9,32	n.a.	46
ML-2	315	522	73	8,46	2,90	66	48
HL-1	72	207	192	4,82	3,51	41	34
HL-2	110	307	123	6,17	3,75	51	38
HL-3	102	208	82	4,26	2,47	42	31
HL-4	107	322	136	6,56	4,43	60	49
HL-5	103	352	167	7,70	5,06	73	61
HL-6*	103	352	167	7,70	5,06	73	61

n.a = not analysed

Table S2. Water quality data for MBBR effluent collected at the day of start-up for each experiment. The unit is mg/l for all parameters.

Experiment	SS	Tot-COD	F-COD	Tot-P	PO4-P	Tot_N	NH4-N
LL-1	206	195	50	6,80	4,68	n.a	17
LL-2	97	n.a	53	8,40	5,99	n.a	8
LL-3	111	175	36	6,52	4,53	56	3
LL-4	158	n.a	19	7,05	6,06	71	9
ML-1	135	n.a	60	4,94	6,42	n.a	43
ML-2	313	451	53	7,44	1,87	51	41
HL-1	96	168	51	4,80	2,21	41	33
HL-2	120	236	66	5,38	3,20	45	36
HL-3	96	175	42	4,02	1,98	37	28
HL-4	171	316	81	6,46	3,68	58	48
HL-5	118	344	122	8,00	4,42	70	55
HL-6*	118	344	122	8,00	4,42	70	55

n.a = not analysed

Table S3. Water quality data for FO feed water. The unit is mg/l for all parameters except pH [-] and Turbidity [NTU].

Experiment	SS	Tot-COD	F-COD	Tot-P	PO4-P	Tot_N	NH4-N	Ca^{2+}	pH	Turbidity
LL-1	36,9	n.a	46,6	4,38	4,30	n.a	16,4	31,22	n.a	17
LL-2	21,9	n.a	51,9	5,93	5,81	n.a	7,99	34,76	n.a	23
LL-3	18,9	54,6	37,6	4,91	4,95	49,30	3,38	56,21	7,2	9
LL-4	13,0	n.a	38,8	5,83	5,87	n.a	8,93	37,16	6,3	7
ML-1	34,9	n.a	75,6	3,56	2,81	n.a	46,35	38,93	n.a	24
ML-2	41,5	90,0	47,1	4,48	3,45	43,90	40	53,91	8,0	29
HL-1	48,1	121,0	45,6	3,61	2,13	37,65	32,2	58,21	n.a	38
HL-2	54,4	130,0	55,3	4,03	2,95	46,20	38,15	57,02	7,9	73
HL-3	29,6	93,2	50,5	2,48	2,10	32,90	29,8	66,45	7,8	22
HL-4	115,6	237,0	77,9	6,00	3,74	55,00	47,6	59,16	8,0	87
HL-5	106,4	292,0	120,0	6,91	4,90	69,80	60,6	44,53	7,9	91
HL-6*	104,7	283,0	111,0	6,45	5,40	72,10	59,60	45,75	5,4	88

n.a = not analysed

Table S4. Water quality data for FO concentrate. The unit is mg/l for all parameters except Turbidity [NTU].

Experiment	Turbidity	Tot-COD	F-COD	Tot-P	PO4-P	Tot_N	NH4-N	Ca^{2+}
LL-1	36	n.a	183	14,2	13,15	0	56	134
LL-2	n.a	n.a	239	29,4	26,40	0	30	157
LL-3	n.a	150	334	40,4	33,33	241	1	440
LL-4	87	n.a	210	30,9	30,01	n.a	n.a	197
ML-1	n.a	n.a	277	7,0	2,83	n.a	185	200
ML-2	243	262	291	15,6	2,91	186	175	154
HL-1	297	246	192	11,2	1,85	141	117	241
HL-2	370	174	264	12,7	4,45	196	155	273
HL-3	344	137	258	8,9	1,98	170	159	262
HL-4	725	984	360	29,0	3,63	325	285	212
HL-5	1762	1370	530	34,0	5,39	351	350	173
HL-6*	495	1930	451	38,5	33,12	407	406	293

n.a = not analysed

3. Water flux vs volumetric concentration factor

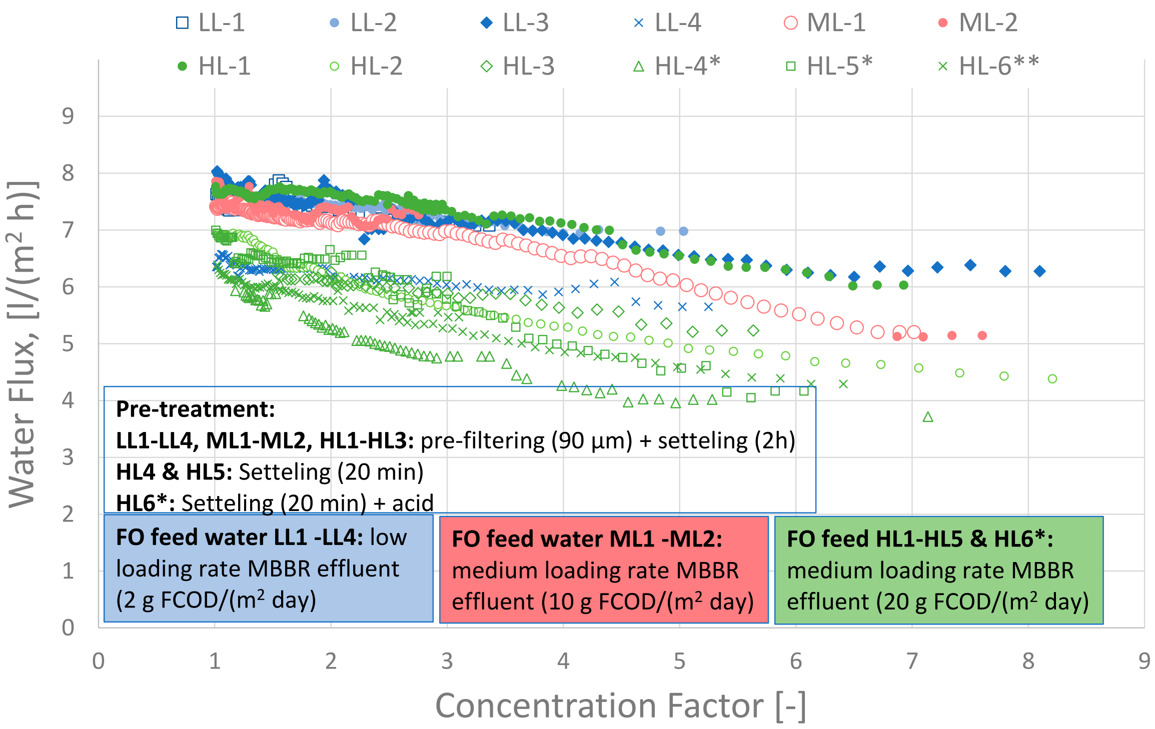

Figure S2 Water flux as a function of volumetric concentration factor measured during period with exposure to M;BBR effluent. Measured fluxes were temperature normalized to 20 °C. The experiments are labelled according to the design value for the biological loading rate of the MBBRs providing the feed water to each of the experiments. *LL* means low biological loading rate, *ML* means medium loading rate, and *HL* means high loading rate.

4. Uncertainty in modelling of baseline flux

As a starting point for the modelling, the characteristic membrane parameters for the applied membrane were determined by modelling of two reference experiments, one in PRO mode (active layer against draw solution) and one in FO mode (active layer against feed side). The following values were obtained; $A = 1.11\cdot10^{-12}$ m/(Pa·s), $B = 2.14\cdot10^{-8}$ m/s and S= 0.32 mm, resulting in a modelled start flux at reference conditions of 6.5 l/(m^2·h), which is in the lower range of the measured start fluxes for the 12 FO experiments.

Due to variations in the water fluxes being measured initially, the characteristic membrane parameters A, B and S should ideally have been determined for each experiment. However, the A, B and S values determined from the reference experiments were applied for the modelling of all the FO experiments. Thus, the modelled water flux was initially underestimated for some of the experiments. However, working with relative flux values, the introduced error in the baseline corrections was not large.

5. Characterization of membrane fouling

5.1 SEM and SEM-EDS

Fouled membrane samples from selected experiments were analyzed by SEM. Selected images are shown in Figure S3. Panel (a) shows a backscatter image (contrast image) of a fouled membrane sample from experiment LL-3. Except from the dust particle in the center of the image a relatively clean membrane surface is observed. However, scattered bright spots indicates minor amounts of inorganic deposits. For Exp. LL-1, LL-2 and LL-4, no deposits were observed. Panel (b) shows a backscatter image of fouled membrane sample from experiment HL-6**. A dense and relatively homogeneous fouling layer was observed. The dark shade indicates predominantly organic content, however, with sparse scattering of bright spots ascribed to inorganic deposits. Panel (c) shows a backscatter image of a fouled membrane sample from experiment HL-5*. It was observed a thick layer of deposits covering the entire membrane surface. The deposits contained numerous relatively large and bright particles unevenly distributed over the membrane surface. Besides the bright particles, the fouling layer seem dense and relatively homogeneous. In panel (d) the large bright particles observed in the backscatter image in panel (c) is shown at 5000 x magnification. The particle appears dumbbell-shaped, and the particle size ranges typically from 1 to 20 μm. Similar observations were made also for membranes from the experiments HL-1, HL-2, HL-3 and HL-4*. However, variation both in number, size and shape of the particles are observed among the different experiments. In general, smaller particles seem to be more linear shaped with less pronounced spherical geometry in each end of the particles.

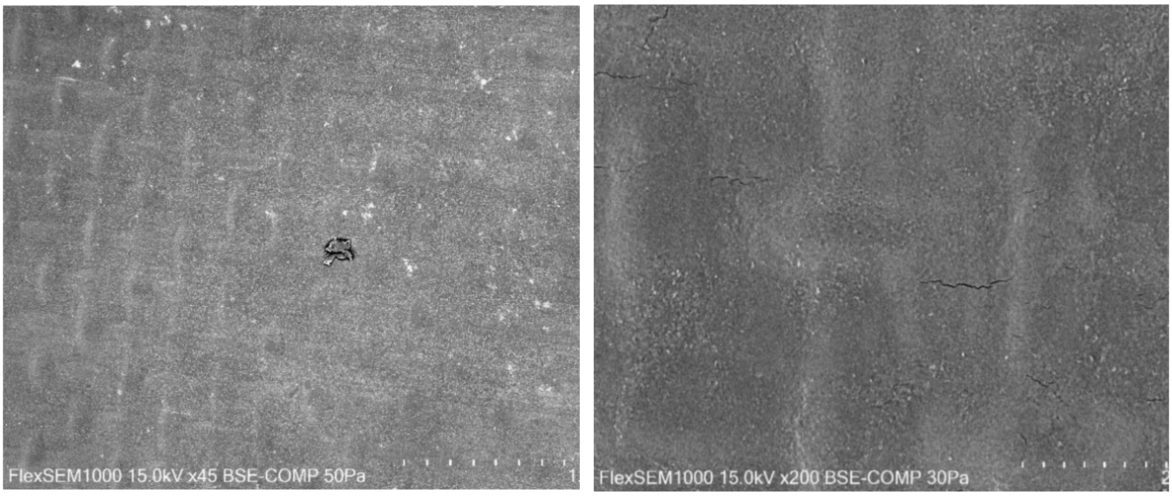

(**a**) (**b**)

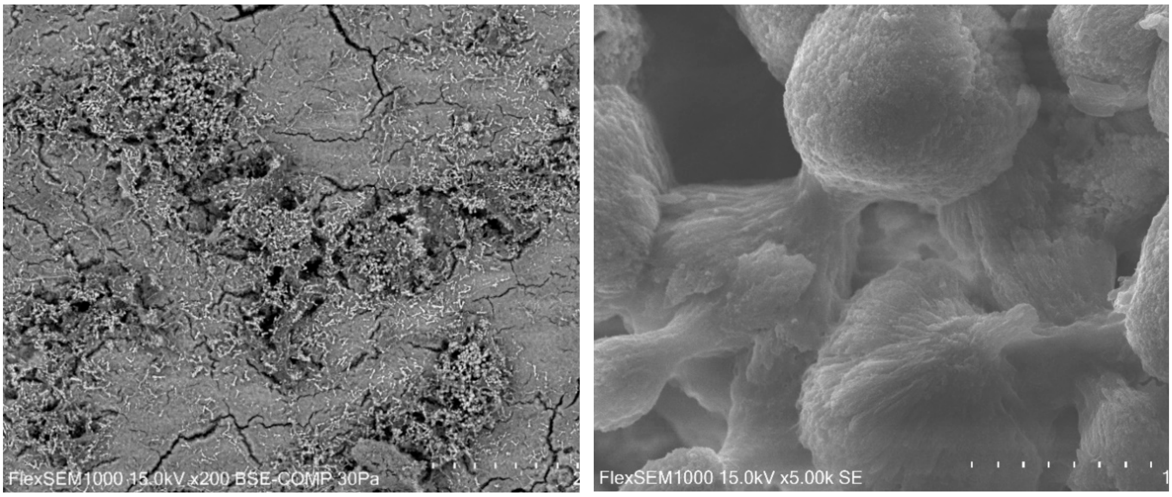

(c) (d)

Figure S3. SEM images of fouled membranes from selected FO experiments. (a) Backscatter image at 45 x magnification of membrane sample from Exp. LL-3; (b); Backscatter image at 200 x magnification of membrane sample from Exp. HL-6** (c) Backscatter image at 200 x magnification of membrane sample from Exp. HL-5*; (d) Secondary electron image at 5000 x magnification of membrane sample from Exp. HL-4*;

Figure S4 presents the results from EDS-analyses of an area with frequent occurrences of larger linear or dumbbell-shaped particles observed as bright spots in backscatter images. Panel (e) in Figure S4 shows a backscatter image with 2000 x magnification of an area with dense deposits and significant occurrence of bright dumbbell-shaped particles in the size range 5-10 µm. Panel (a), (b) (c) and (d) presents the signal plots for Ca, P, O and C, respectively, which has been obtained by EDS-analyses. The relative occurrence of the respective elements is given as mol% in the table in panel (f). The areas corresponding to the larger linear or dumbbell-shaped particles has a strong signal for C, O and Ca, whereas the signal for P is absent. The observations indicate that the particles consist of $CaCO_3$. EDS-analyses of small areas only covering a selected particle shows a relative molar composition of C:O:Ca corresponding to 22:60:16. For comparison the theoretical molar composition of $CaCO_3$ is 20:60:20.

The areas surrounding the larger particles has a relative uniform signal for all four elements C, O, Ca and P. A carbon signal of almost 50% indicates significant organic composition. However, the Ca and P signal indicates partly inorganic composition as well. No other elements were found to a significant extent, thus, the inorganic component of the uniform deposits is assumed to be $CaHPO_4$.

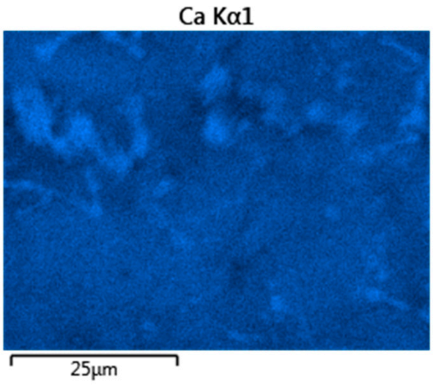

(a)

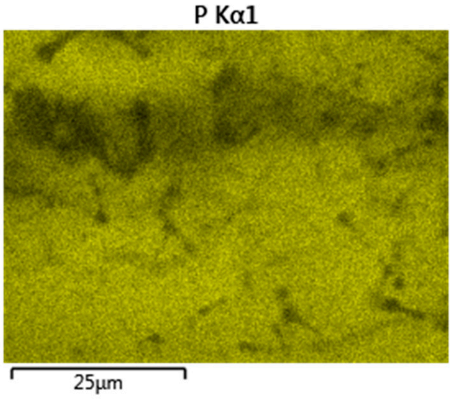

(b)

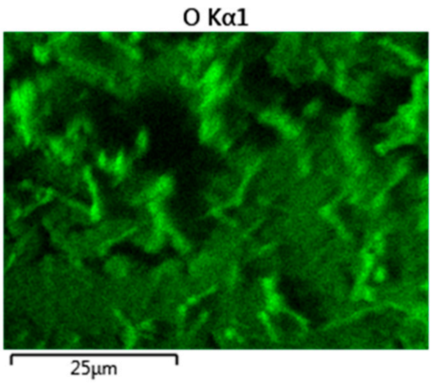

(c)

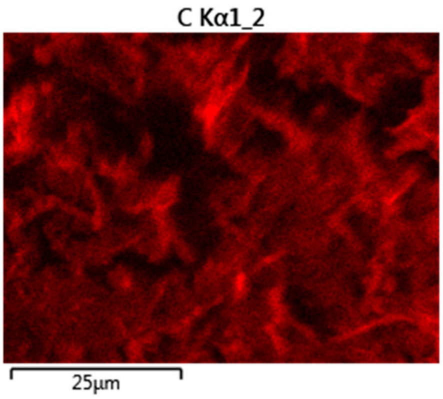

(d)

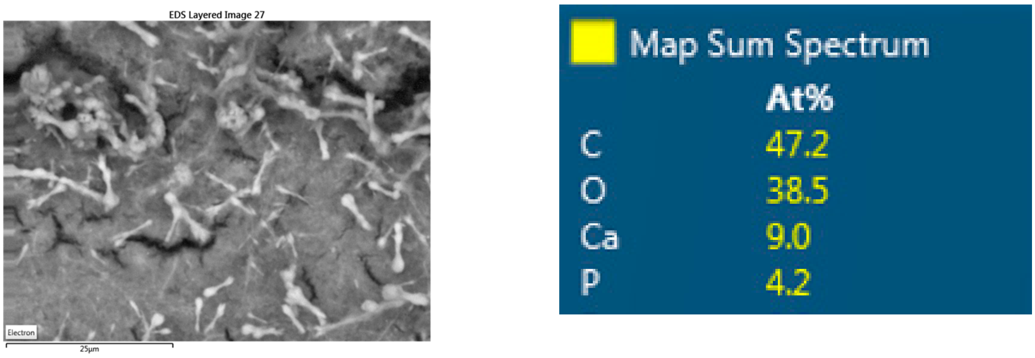

(e) (f)

Figure S4. SEM-EDS analyses of deposits on membrane from Exp. HL-5*. (a) Calcium signal; (b) Phosphorous signal; (c) Oxygen signal; (d) Carbon signal; (e) Backscatter image at 2000 x magnification; and (f) relative molar composition [mol%].

Figure 5 presents the ratios for C/O and Ca/P in deposits obtained by EDS analyses. The EDS analyses is based on relatively large areas, in the range of 0.1 mm x 0.1 mm for HL-1 and HL-2, and 0.3 mm x 0.3 mm for the others. The intention was to cover a large enough area to omit effects of inhomogeneities, such as unevenly distributed clusters of large $CaCO_3$ particles. The C/O ratio is related to the relative share of organic versus inorganic content of the deposits. A C/O ratio of 2.5, which is observed for HL-6**, is typical for natural organic matter and indicates low or no inorganic content. As a contrast, a C/O ratio of 0.33 would indicate pure CaCO3. Thus, the results indicate a predominantly inorganic content for the experiments HL-1, HL-2, HL-3 and HL-4*. Note that the C/O ratio reported for LL-3 and LL-4 relates to the membrane polymer since no or very little deposits were present on membranes from those experiments.

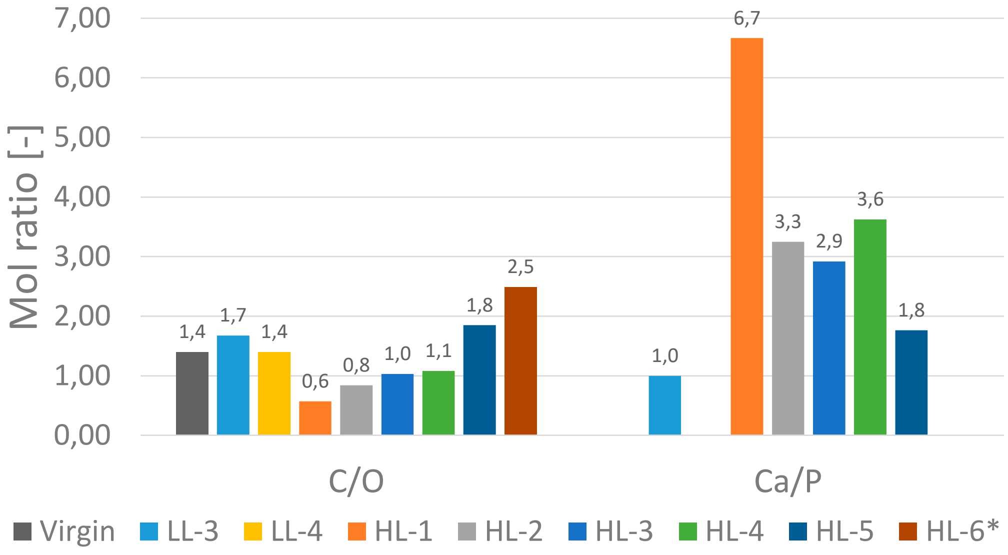

Figure 5. Molar ratios for carbon/oxygen and calcium/phosphorous obtained by EDS-analyses on fouled membranes from FO experiments. For LL-4 and HL-6** the Ca/P ratio was not obtained due to insignificant content of inorganic material.

The Ca/P ratio is a measure of the relative contribution of $CaCO_3$ vs $CaHPO_4$ in the fouling deposits. The higher ratio the higher share of $CaCO_3$. A ratio of 1, which is observed for LL-3 indicates that the entire inorganic content is ascribed to $CaHPO_4$. For LL-4 and HL-6** the Ca/P ratio was not obtained due to insignificant content of inorganic material in deposits.

The results for HL-1 are not believed to be representative due the selected area for EDS analyses corresponded to a location with large accumulation of $CaCO_3$ particles. As a result, the C/O ratio is underestimated, and the Ca/P ratio is overestimated for HL-1.

5.2 Characterization of dissolved deposits

The composition of dissolved fouling deposits scrapped off membranes from selected experiments was analyzed by ICP-MS. The results are shown in Figure S6.

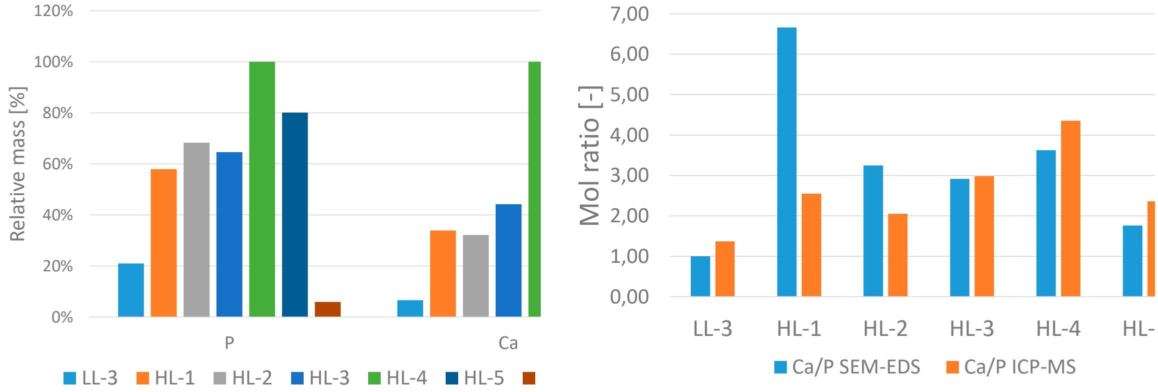

(a) **(b)**

Figure S6. (a) Mass of P and Ca obtained from ICP-MS of dissolved fouling deposits. 100% refers to the sample with highest mass (HL-4*); (b) Molar Ca/P ratio obtained from ICP-MS of dissolved fouling deposits and SEM-EDS of fouled membranes, respectively.

Panel (a) in Figure S6 presents the mass of Ca and P in dissolved deposits. Exp. HL-4* contained the highest mass of Ca and P and was therefore used as a relative reference, being set to 100%. The mass of inorganic precipitate was observed to be relatively high also for the experiments HL-1, HL-2, HL-3 and HL-5*. For experiments LL-3 and HL-6** the mass of inorganic precipitate was low compared to the other experiments. Panel (b) shows the molar ratio of Ca/P in dissolved deposits determined by ICP-MS, and the Ca/P ratio determined on fouled membranes by EDS analyses. The latter is the same results as presented in Figure 5. Except from experiment HL-1, the results from the two different calculations are consistent. Due to the unintended use of a non-representative sampling area for the EDS-analyses performed for experiment HL-6, the Ca/P ratio of 2.6 determined by ICP-MS is believed to be more correct.

6. Explanation of variables included in PCA

Table S5. Explanation of variables included in the PCA.

Short name	Explaination	Category	Unit
Particulate_P	Relative share of phosphorous in FO feed found as particles	Feed parameter	%
Particulate_COD	Relative share of COD in FO feed found as particles	Feed parameter	%
NH4_N_feed	Concentration of NH4_N in FO feed	Feed parameter	mg/l
Alk_feed	Alkalinity in FO feed	Feed parameter	meq./l
Turbidity_feed	Turbidity in FO feed	Feed parameter	NTU
pH_feed	pH in FO feed	Feed parameter	-
Nitrification	Degree of nitrification in MBBR	Feed parameter	%
Accum_COD_load	Accumulated SS loading per membrane area		mg COD/m^2
Accum_SS_load	Accumulated COD loading per membrane area		mg SS/m^2
Final_Flux	Relative baseline-corrected flux at the end of the experiment	Response	%
CF_PO4_P	Concentration factor of PO4-P	Response	-
CF_Ca	Concentration factor of Ca^{2+}	Response	-
Mass_P_deposits	Specific mass of P in membrane deposit relative to exp. highest value	Response	%
Mass_Ca_deposits	Specific mass of Ca in membrane deposit relative to exp. highest value	Response	%
Molratio_C_O	Mol ratio Ca/P in fouling deposits	Response	-
Molratio_Ca_P	Mol ratio C/O in fouling deposits	Response	-
